# Supplementary material for: In Vitro Heme Coordination of a Dye-Decolorizing Peroxidase—The Interplay of Key Amino Acids, pH, Buffer and Glycerol
Source: Int J Mol Sci. 2021 Sep 12;22(18):9849. doi: 10.3390/ijms22189849 (PMC8468270; doi:10.3390/ijms22189849)
Supplement: Supplementary file 1 [file ijms-22-09849-s001.zip › ijms-1302052-supplementary.pdf]

Supplementary Material to

In vitro heme coordination of a dye-decolorizing peroxidase - the interplay of key amino acids, pH, buffer and glycerol

Kevin Nys[1], Vera Pfanzagl[2], Jeroen Roefs[1], Christian Obinger[2] and Sabine Van Doorslaer[1,*]

[1] BIMEF Laboratory, Department of Chemistry, University of Antwerp, Antwerp, Belgium
[2] Division of Biochemistry, Department of Chemistry, BOKU – University of Natural Resources and Life Sciences, Vienna, Austria
* Correspondence: sabine.vandoorslaer@uantwerpen.be

CONTENT

1. Field-swept EPR experiments
   1.1. Experimental data
   1.2. Simulation parameters
2. HYSCORE spectroscopy
3. References

1. Field-swept EPR

1.1. Experimental data

Figure S1

X-band CW EPR spectra of frozen solutions of WT *Kp*DyP and variants in phosphate buffer (pH 7.0) without (A) and with (B) glycerol. Experimental (black) and simulated (red) spectra are given with the different contributions as dashed-dotted lines. The simulation data are given in Table S1. An asterisk indicates the position of a stable organic radical identified earlier in the resting-state of the protein [1]. The spectra are shown normalized to allow facile comparison. The color code of the simulated spectra is as shown on top of the figure.

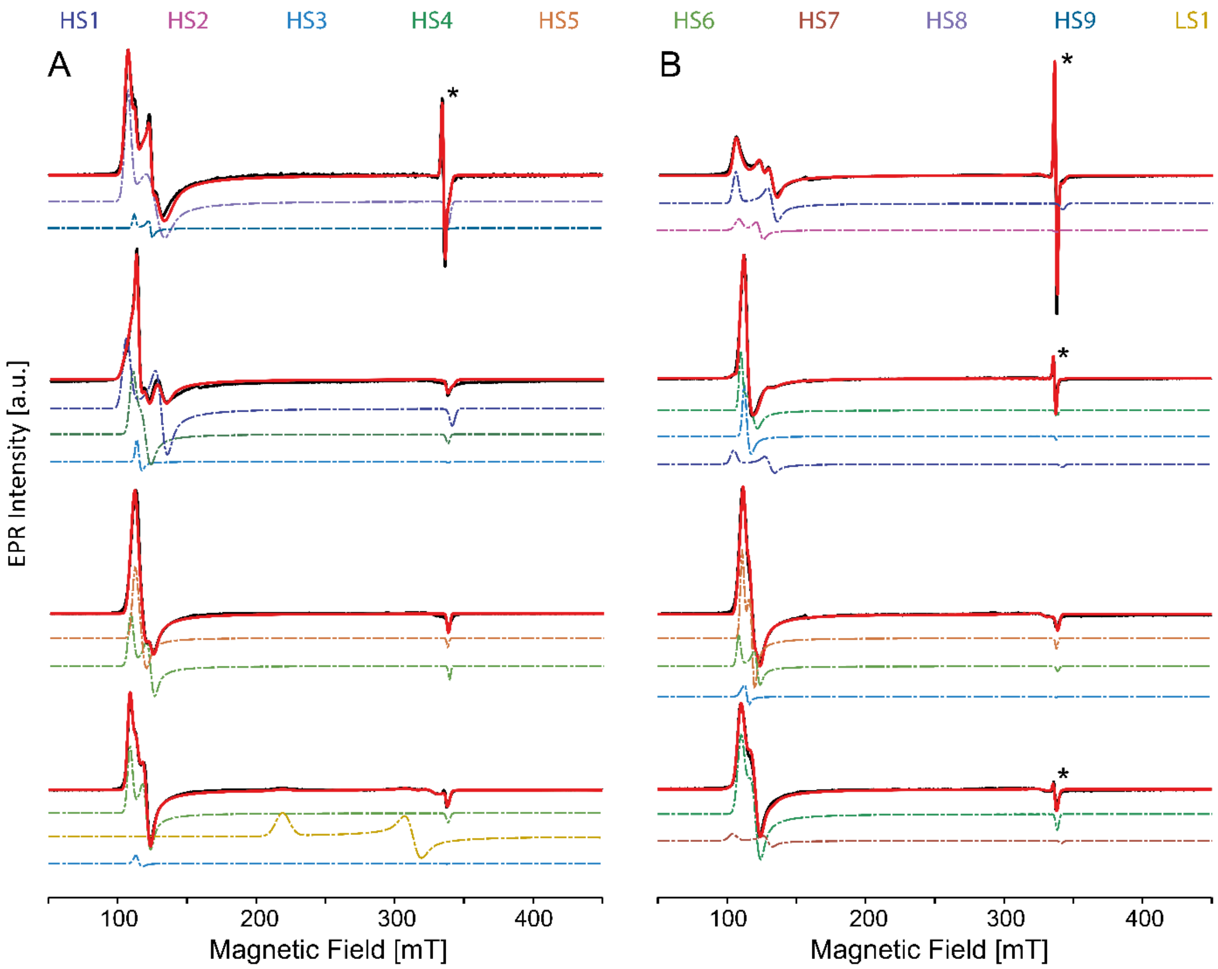

Figure S2

X-band CW EPR spectra of frozen solutions of WT *Kp*DyP and variants in borate buffer (pH 10.0) without (A) and with (B) glycerol. Experimental (black) and simulated (red) spectra are given with the different contributions as dashed-dotted lines. The simulation data are given in Table S2. An asterisk indicates the position of a stable organic radical identified earlier in the resting-state of the protein [1]. The spectra are shown normalized to allow facile comparison. The color code of the simulated spectra is as shown on top of the figure. Figure S3 shows an enlargement in the region of the LS contributions.

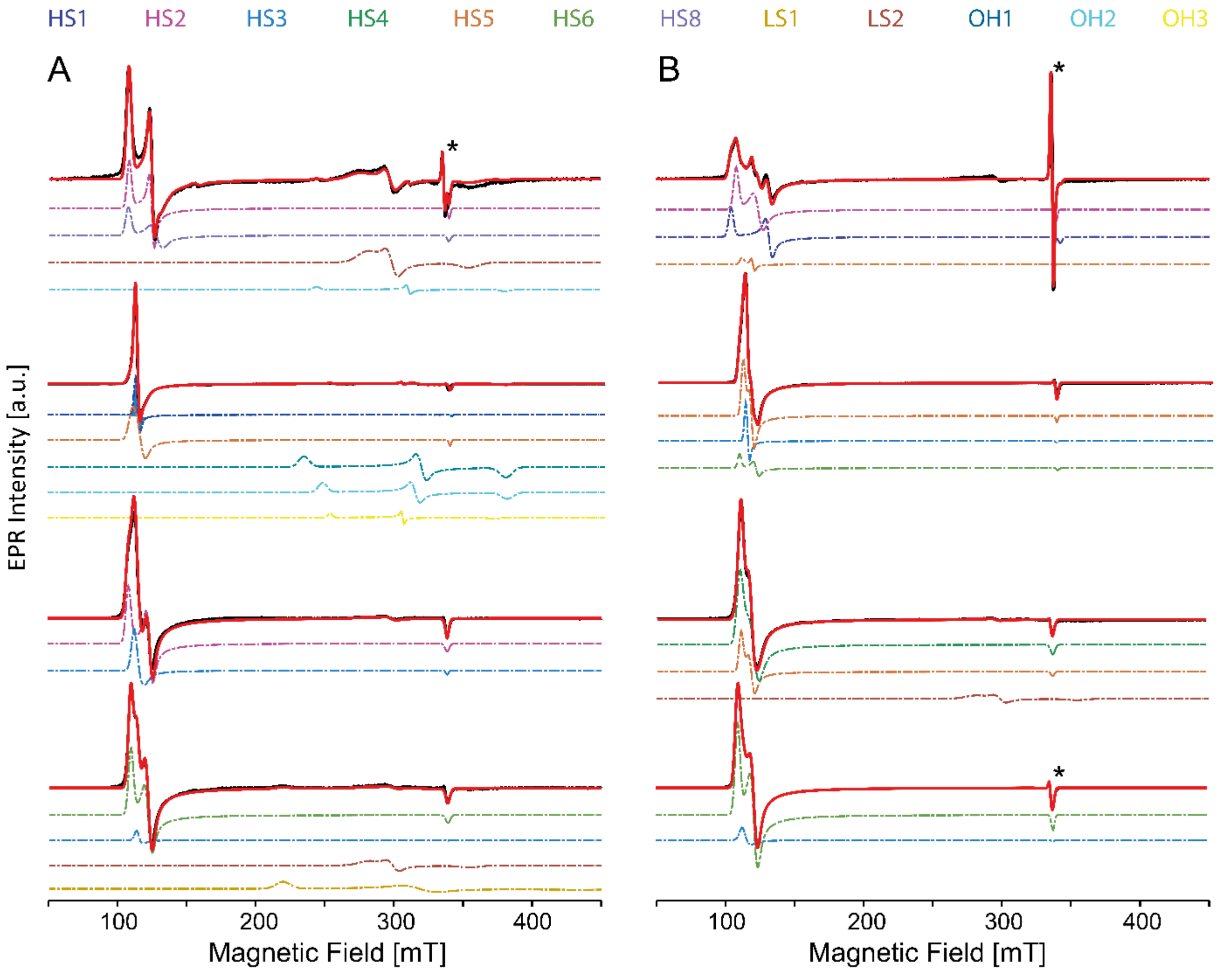

Figure S3

X-band CW EPR spectra of frozen solutions of WT *Kp*DyP and variants in borate buffer (pH 10.0) without (A) and with (B) glycerol. Experimental (black) and simulated (red) spectra are given in the range where LS Fe(III) contributions are expected (detail of Figure S2). An asterisk indicates the position of a stable organic radical identified earlier in the resting-state of the protein [1]. The spectra are shown normalized to allow facile comparison and the g_z-component of the OH1′ (dashed-dotted), OH2/OH2′ (dashed) and OH3′ (dotted) species is indicated. The simulation data are given in Table S2.

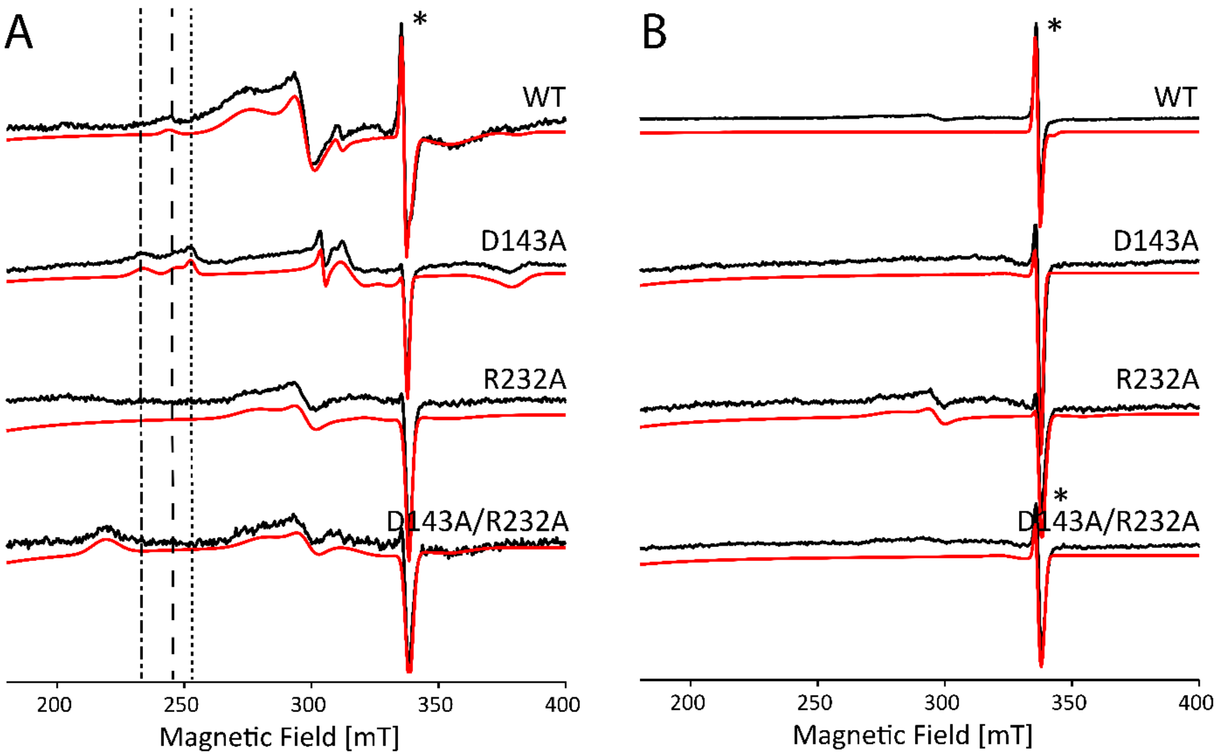

Figure S4

X-band CW EPR spectra of frozen solutions of WT *Kp*DyP and variants in glycine-KOH buffer (pH 10.0) without (A) and with (B) glycerol. Experimental (black) and simulated (red) spectra are given with the different contributions as dashed-dotted lines. The simulation data are given in Table S3. An asterisk indicates the position of a stable organic radical identified earlier in the resting-state of the protein [1]. The spectra are shown normalized to allow facile comparison. The color code of the simulated spectra is as shown on top of the figure.

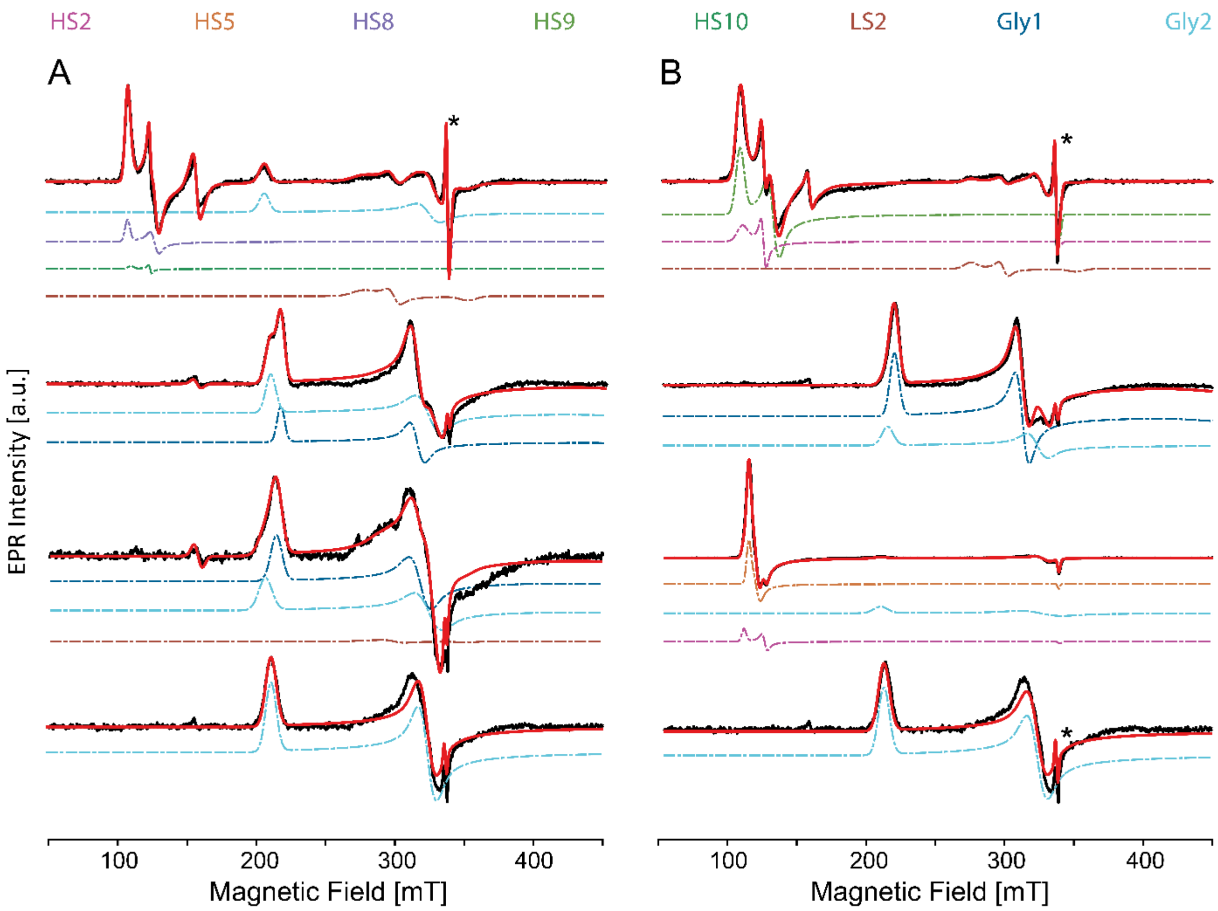

Figure S5

X-band CW EPR spectra of frozen solutions of WT *Kp*DyP and variants in glycine-KOH buffer (pH 10.0) without (A) and with (B) glycerol. Experimental (black) and simulated (red) spectra are given in the range where LS Fe(III) contributions are expected (detail of Figures S4). An asterisk indicates the position of a stable organic radical identified earlier in the resting-state of the protein [1]. The spectra are shown normalized to allow facile comparison and the g_z-component of the Gly1 (dashed-dotted) and Gly2/Gly2′ (dashed) species is indicated.

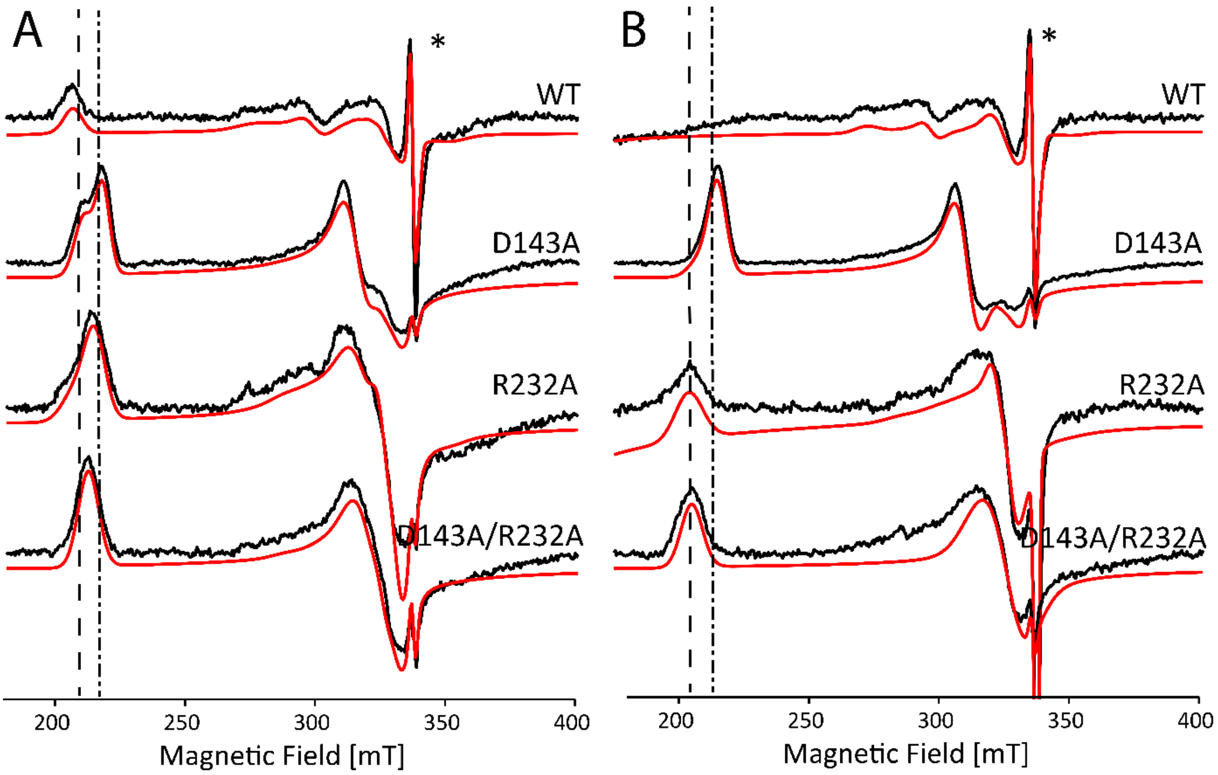

Figure S6

W-band electron spin echo (ESE)-detected EPR spectra of frozen solutions of WT *Kp*DyP (A) and three variants: (B) D143A, (C) R232A and (D) D143A/R232A in glycine-KOH buffer (pH 10.0). Inter-pulse time (τ) equals 200 ns for the low-field part and τ = 400 ns for the high-field spectrum. All spectra suffer from a Mn(II) background indicated by an asterisk.

The spectra confirm the earlier observation in Figure 6B (main text) that HS forms are present in frozen solutions of WT and R232A *Kp*DyP, but not for the two other variants. The spectra also allowed for a confirmation of the EPR parameters of the HS forms and an accurate determination of the g_z values of the Gly2-type components (dashed line). However, the weak electron spin echo intensity and the presence of background signals due to contaminations (Cu(II) and Mn(II)) prevented an improved determination of the other principal g values of this species.

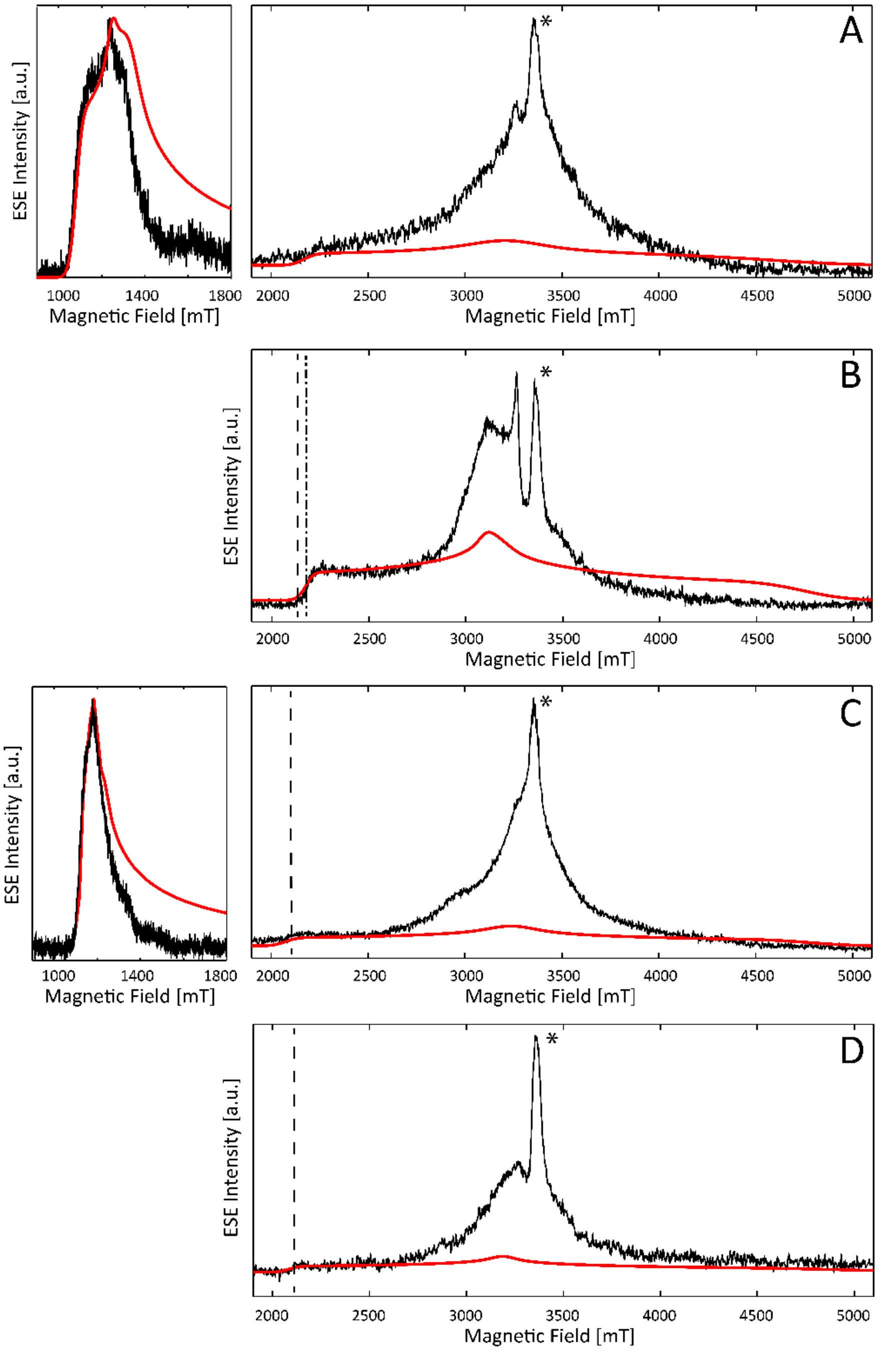

1.2. Experimental simulation parameters

In Tables S1-S3 the simulation parameters of the experimental spectra in Figures 3, 5 and 6 (main text) and Figures S1-S6 (supplementary information) are given. Simulations of the HS Fe(III) species were done in two ways: assuming an effective S = 1/2 system with effective g-values (experimental error: ± 0.01 for g_z^{eff} and ± 0.02 for $g_{x,y}^{eff}$) and assuming an S = 5/2 system with g-values close to 2 (experimental error: ± 0.005) and varying E/D values (with D and E being the tetragonal and rhombic zero-field splitting, respectively). In this case, only the E/D ratio can be determined (experimental error: ± 0.001), where E is a measure for the rhombicity of the zero-field tensor. D is taken to be equal to 9.26 cm^{-1}, the value found for aquometmyoglobin [2]. As long as the D value is significantly higher than the microwave quantum, the specific value of D does not affect the spectra, only the E/D value does. The relative contributions of the species were determined using the S = 5/2 simulation method (experimental error ± 1%).

Table S1

EPR simulation parameters of frozen solution spectra from WT *Kp*DyP and variants dissolved in phosphate buffer (pH 7.0) with and without glassing agent (Figure 3, main text). R = radical.

		g_x^{eff}	g_y^{eff}	g_z^{eff}	g_x	g_y	g_z	*E/D*	*I (%)*
with glycerol [1]									
**WT**									
	HS1	6.44	5.12	1.98	1.93	1.93	2.00	0.0288	77.3
	HS2	6.25	5.41	1.99	1.95	1.95	2.00	0.0178	22
	R[a)]				~2	~2	~2		0.7
**D143A**									
	HS3	5.99	5.85	2.00	1.97	1.97	2.00	0.0029	40.7
	HS4	6.13	5.69	1.98	1.97	1.97	2.00	0.0097	48.2
	HS1	6.43	5.15	1.97	1.94	1.94	2.00	0.0279	11.1
**R232A**									
	HS5	6.06	5.69	1.99	1.96	1.96	2.00	0.0076	67.2
	HS6	6.2	5.52	1.99	1.95	1.95	2.00	0.0148	23.9
	HS3	6.05	5.88	2.00	1.99	1.99	2.00	0.0036	8.9
**D143A/R232A**									
	HS4	6.13	5.61	1.99	1.96	1.96	2.00	0.0086	91.7
	HS7	6.49	5.21	1.98	1.96	1.96	2.00	0.0220	8.3
without glycerol									
**WT**									
	HS8	6.23	5.26	2.00	1.92	1.92	2.00	0.0216	88.2
	HS9	5.97	5.402	1.99	1.90	1.90	2.00	0.0125	11
	R[a)]				~2	~2	~2		0.8
**D143A**									
	HS1	6.4	5.16	1.98	1.93	1.93	2.00	0.0270	45.3
	HS4	6.13	5.65	2.00	1.96	1.96	2.00	0.0104	40.7
	HS3	5.96	5.89	2.00	1.97	1.97	2.00	0.0022	14
**R232A**									
	HS5	6.04	5.77	1.99	1.97	1.97	2.00	0.006	57.1
	HS6	6.22	5.49	2.00	1.96	1.96	2.00	0.0156	42.9
**D143A/R232A**									
	HS6	6.19	5.57	1.99	1.96	1.96	2.00	0.0133	68
	HS3	5.95	5.85	1.98	1.97	1.97	2.00	0.0029	8
	LS1				1.40	2.15	3.076		24

[a)] *g*-values of radical are approximated– detailed analysis is subject of different study

Table S2

EPR simulation parameters of frozen solution spectra from WT *Kp*DyP and variants dissolved in borate buffer (pH 10.0) with and without glassing agent.

†*g*-value theoretically predicted using $g_x^2+g_y^2+g_z^2 = 16$ [S3], R, radical.

		g_x^{eff}	g_y^{eff}	g_z^{eff}	g_x	g_y	g_z	*E/D*	*I (%)*
with glycerol									
**WT**									
	HS2	6.24	5.43	1.99	1.95	1.95	2.00	0.0174	47.9
	HS1	6.47	5.12	1.97	1.94	1.94	2.00	0.0295	43.1
	HS5	6.01	5.61	2.00	1.94	1.94	2.00	0.0086	5.7
	R[a)]				~2	~2	~2		3.3
**D143A**									
	HS5	6.07	5.74	2.00	1.97	1.97	2.00	0.0068	51.4
	HS3	5.97	5.88	2.00	1.98	1.98	2.00	0.0017	35
	HS6	6.21	5.59	1.99	1.97	1.97	2.00	0.0132	13.6
**R232A**									
	HS4	6.12	5.6	1.99	1.95	1.95	2.00	0.0111	62.2
	HS5	6.08	5.68	2.00	1.96	1.96	2.00	0.0084	33.9
	LS2				1.90	2.28	2.42		3.9
**D143A/R232A**									
	HS6	6.17	5.57	1.99	1.96	1.96	2.00	0.0129	87.7
	HS3	5.96	5.82	1.99	1.97	1.97	2.00	0.0025	12.3
without glycerol									
**WT**									
	HS2	6.24	5.42	1.98	1.95	1.95	2.00	0.0170	41.2
	HS8	6.28	5.26	1.98	1.93	1.93	2.00	0.0222	24.9
	OH2				1.77	2.17	2.77		4.2
	LS2				1.90	2.27	2.46		29.1
	R[a)]				~2	~2	~2		0.6
**D143A**									
	HS3	5.95	5.91	2.00	1.98	1.98	2.00	0.0013	36.8
	HS5	6.11	5.76	1.99	1.98	1.98	2.00	0.0074	33.4
	OH1′				1.78	2.12	2.89		13.4
	OH2′				1.775	2.15	2.728		10
	OH3′				1.82	2.214	2.67		6.4
**R232A**									
	HS2	6.24	5.46	1.99	1.95	1.95	2.00	0.0166	54.4
	HS3	5.98	5.8	1.99	1.96	1.96	2.00	0.004	39.1
	LS2				1.90	2.27	2.43		6.5
**D143A/R232A**									
	HS6	6.2	5.55	1.99	1.96	1.96	2.00	0.0141	63.7
	HS3	5.95	5.85	1.99	1.97	1.97	2.00	0.0022	9.2
	LS1				1.46†	2.11	3.07		22.3
	LS2				1.90	2.26	2.41		4.8

[a)]*g*-values of radical are approximated – detailed analysis is subject of different study

Table S3

EPR simulation parameters of frozen solution spectra from WT *Kp*DyP and variants dissolved in glycine-KOH buffer (pH 10.0) with and without glassing agent.

† *g*-value theoretically determined using $g_x^2+g_y^2+g_z^2 = 16$ [3], R= radical.

		g_x^{eff}	g_y^{eff}	g_z^{eff}	g_x	g_y	g_z	*E/D*	*I (%)*
with glycerol									
**WT**									
	HS9	6.3	5.14	1.99	1.91	1.91	2.00	0.0255	68.9
	HS2	6.22	5.44	1.99	1.95	1.95	2.00	0.0168	23.6
	LS2				1.92	2.26	2.46		6.9
	R[a)]				~2	~2	~2		0.6
**D143A**									
	Gly1				1.41	2.16	3.08		76.9
	Gly2				1.28†	2.09	3.16		23
**R232A**									
	HS2	6.23	5.46	1.99	1.95	1.95	2.00	0.0164	13.2
	HS5	6.02	5.74	2.00	1.96	1.96	2.00	0.0061	45.5
	Gly2				1.10†	2.07	3.24		41.3
**D143A/R232A**									
	Gly2				1.12†	2.09	3.22		100
**without glycerol**									
**WT**									
	HS8	6.24	5.29	1.99	1.93	1.93	2.00	0.0207	23
	HS10	6.11	5.43	1.99	1.93	1.93	2.00	0.0148	4.1
	Gly2				1.00†	2.08	3.27		64.3
	LS2				1.92	2.27	2.45		8.3
	R[a)]				~2	~2	~2		0.3
**D143A**									
	Gly2				1.15†	2.09	3.21		51.5
	Gly1				1.40	2.14	3.11		48.5
**R232A**									
	Gly1				1.30†	2.11	3.14		58.1
	Gly2				1.04†	2.07	3.26		40.7
	LS2				1.92	2.26	2.41		1.9
**D143A/R232A**									
	Gly2′				1.22†	2.10	3.18		100

[a)] *g*-values of radical are approximated – detailed analysis is subject of different study

Table S4

Overview of the different contributing spin systems, observed in low-temperature CW-EPR spectroscopy. The table shows the influence of changing pH, buffer and the addition of glycerol.

	**phosphate pH 7.0**		**borate pH 10.0**		**glycine KOH pH 10.0**	
	**no glycerol**	**glycerol**	**no glycerol**	**glycerol**	**no glycerol**	**glycerol**
**WT**	HS8<br>HS9	HS1<br>HS2	HS2 OH2<br>HS8 LS2	HS1<br>HS2<br>HS5	HS8 Gly2<br>HS10 LS2	HS2 LS2<br>HS9
**D143A**	HS1<br>HS3<br>HS4	HS1<br>HS3<br>HS4	HS3 OH1′<br>HS5 OH2′<br>OH3′	HS3<br>HS5<br>HS6	Gly1<br>Gly2	Gly1<br>Gly2
**R232A**	HS5<br>HS6	HS3<br>HS5<br>HS6	HS2 LS2<br>HS3	HS4 LS2<br>HS5	Gly1<br>Gly2<br>LS2	HS2 Gly2<br>HS5
**D143A/ R232A**	HS3 LS1<br>HS6	HS4<br>HS7	HS3 LS1<br>HS6 LS2	HS3<br>HS6	Gly2′	Gly2

2. HYSCORE spectroscopy

Figure S7

^{14}N HYSCORE of WT *Kp*DyP shows signals of strongly coupled nuclei in the (-,+) quadrant (right figure). The energy scheme (left) shows the consecutive splittings caused by the electron Zeeman and nuclear Zeeman interaction and a shift in energy levels because of the hyperfine interaction. For a ^{14}N nucleus , the coupling with the unpaired electron of the Fe(III) leads to four single-quantum transitions (sq, $|\Delta m_S|$= 0, $|\Delta m_I|$ = 1, red) and two double-quantum transitions (dq, $|\Delta m_S|$ = 0, $|\Delta m_I|$ = 2, blue). Combination of these frequencies leads to the cross peaks in the HYSCORE spectrum as indicated in the spectrum of *Kp*DyP (right).

Spectral simulation of cross peaks reminiscent of the porphyrin ^{14}N (Figure 7) was performed using the Easyspin function *saffron*. Since it consists of HYSCORE spectra at g ≈ 2, where the zero-field splitting has limited effect, we used an effective S^{eff} = ½ system with the g^{eff}-tensor determined in [1]. While A_z^{eff} values were determined experimentally (Table 2), the $A_{x,y}^{eff}$ values were estimated based on the work on aquometmyoglobin [4], taking into account the second-order approximation for the effective spin-Hamiltonian described before [2].

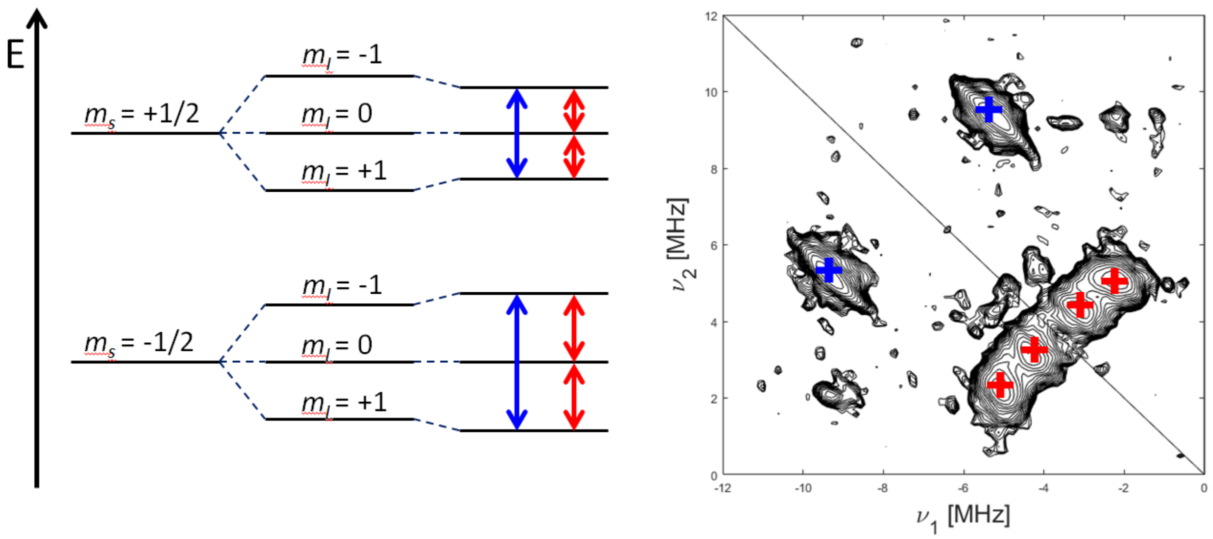

Figure S8

(+,+) quadrant of the X-band matched HYSCORE spectrum of a frozen solution of WT *Kp*DyP taken at 4 K and magnetic-field setting B_0 = 136 mT (corresponding to g_y^{eff}). Experimental spectrum (black) and spectral simulations assuming the same ^{1}H hyperfine values of the protons at the C_ε and C_δ positions of the proximal His (red) and mesoprotons (green) in aquometmyoglobin [5]. The clear match confirms the assignment of these signals and similar proximal His ligation.

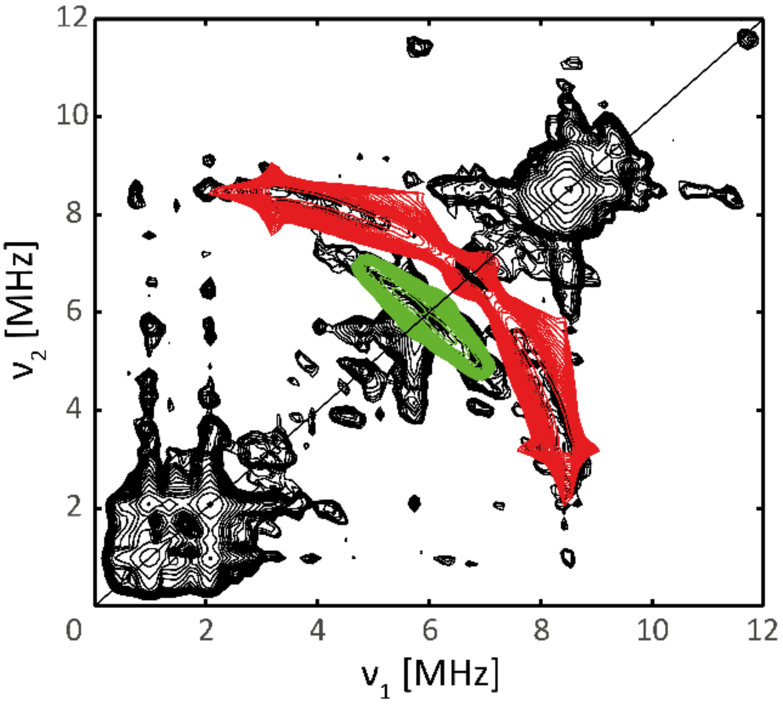

Figure S9

Lower frequency area ([0-7; 0-7] MHz) of X-band HYSCORE of a frozen solution of *Kp*DyP variants taken at 4 K and magnetic-field setting corresponding to $g = g_z^{eff}$. (a) HYSCORE spectrum of WT *Kp*DyP – This area of the HYSCORE spectra includes the contributions of weakly coupled ^{14}N nuclei (dashed-dotted circle), such as the N_δ of the proximal histidine [S4] and the distal Arg-232, and the remote ^{13}C nuclei (the anti-diagonal through the ^{13}C Larmor frequency is indicated with dashed lines).

Comparison between the HYSCORE spectra of WT (black), R232A (blue) and D143A (green) *Kp*DyP. A clear upshift of the HYSCORE cross-peaks due to the weakly coupled ^{14}N nuclei can be observed for the R232A *Kp*DyP variant, linked to the mutation of the Arg-232. Mutation of the Asp-143 does not have this effect.

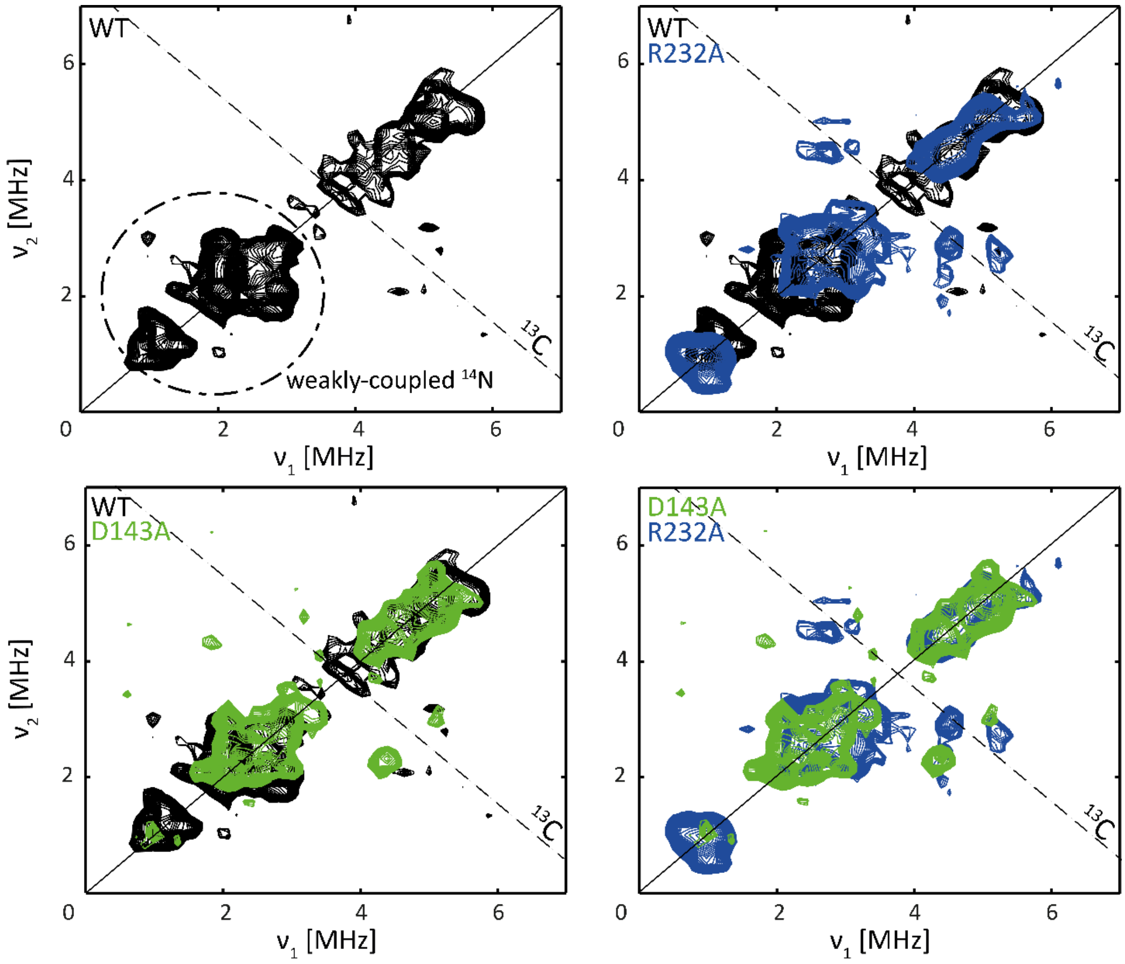

3. References

1. Pfanzagl, V.; Nys, K.; Bellei, M.; Michlits, H.; Mlynek, G.; Battistuzzi, G.; Djinovic-Carugo, K.; Van Doorslaer, S.; Furtmüller, P. G.; Hofbauer, S., Roles of distal aspartate and arginine of B-class dye-decolorizing peroxidase in heterolytic hydrogen peroxide cleavage. *Journal of Biological Chemistry* **2018,** 293, (38), 14823-14838, 10.1074/jbc.RA118.004773.
2. Scholes, C. P.; Lapidot, A.; Mascarenhas, R.; Inubushi, T.; Isaacson, R. A.; Feher, G., Electron nuclear double resonance (ENDOR) from heme and histidine nitrogens in single crystals of aquometmyoglobin. *Journal of the American Chemical Society* **1982,** 104, (10), 2724-2735, DOI 10.1021/ja00374a007.
3. Walker, F. A., Magnetic spectroscopic (EPR, ESEEM, Mössbauer, MCD and NMR) studies of low-spin ferriheme centers and their corresponding heme proteins. *Coordination Chemistry Reviews* **1999,** 185-186, 471-534, https://doi.org/10.1016/S0010-8545(99)00029-6.
4. Fittipaldi, M.; García-Rubio, I.; Trandafir, F.; Gromov, I.; Schweiger, A.; Bouwen, A.; Van Doorslaer, S., A multi-frequency pulse EPR and ENDOR approach to study strongly coupled nuclei in frozen solutions of high-spin ferric heme proteins. *The Journal of Physical Chemistry B* **2008,** 112, (12), 3859-3870, 10.1021/jp709854x.
5. García-Rubio, I.; Fittipaldi, M.; Trandafir, F.; Van Doorslaer, S., A multifrequency HYSCORE study of weakly coupled nuclei in frozen solutions of high-spin aquometmyoglobin. *Inorganic chemistry* **2008,** 47, (23), 11294-11304, 10.1021/ic8016886.
